# Mathematical Modeling and Optimization of *Lactobacillus* Species Single and Co-Culture Fermentation Processes in Wheat and Soy Dough Mixtures

**DOI:** 10.3389/fbioe.2022.888827

**Published:** 2022-06-23

**Authors:** Eva-H. Dulf, Dan C. Vodnar, Alex Danku, Adrian Gheorghe Martău, Bernadette-Emőke Teleky, Francisc V. Dulf, Mohamed Fawzy Ramadan, Ovidiu Crisan

**Affiliations:** ^1^ Faculty of Automation and Computer Science, Technical University of Cluj-Napoca, Cluj-Napoca, Romania; ^2^ Institute of Life Sciences, University of Agricultural Sciences and Veterinary Medicine, Cluj-Napoca, Romania; ^3^ Faculty of Food Science and Technology, Institute of Life Sciences, University of Agricultural Sciences and Veterinary Medicine of Cluj-Napoca, Cluj-Napoca, Romania; ^4^ Faculty of Agriculture, University of Agricultural Sciences and Veterinary Medicine Cluj-Napoca, Cluj-Napoca, Romania; ^5^ Deanship of Scientific Research, Umm Al-Qura University, Makkah, Saudi Arabia; ^6^ Department of Agricultural Biochemistry, Faculty of Agriculture, Zagazig University, Zagazig, Egypt; ^7^ Department of Organic Chemistry, Iuliu Hațieganu University of Medicine and Pharmacy, Cluj-Napoca, Romania

**Keywords:** lactic acid bacteria, process optimization, mathematical model, regression, artificial neural network (ANN)

## Abstract

To improve food production *via* fermentation with co-cultures of microorganisms (e.g., multiple lactic acid bacteria-LAB strains), one must fully understand their metabolism and interaction patterns in various conditions. For example, LAB can bring added quality to bread by releasing several bioactive compounds when adding soy flour to wheat flour, thus revealing the great potential for functional food development. In the present work, the fermentation of three soy and wheat flour mixtures is studied using single cultures and co-cultures of *Lactobacillus plantarum* and *Lactobacillus casei*. Bio-chemical processes often require a significant amount of time to obtain the optimal amount of final product; creating a mathematical model can gain important information and aids in the optimization of the process. Consequently, mathematical modeling is used to optimize the fermentation process by following these LAB’s growth kinetics and viability. The present work uses both multiple regression and artificial neural networks (ANN) to obtain the necessary mathematical model, useful in both prediction and process optimization. The main objective is to find a model with optimal performances, evaluated using an ANOVA test. To validate each obtained model, the simulation results are compared with the experimental data.

## 1 Introduction

Humans’ survival and disease risk is mainly conditioned by lifestyle, genetic, and environmental factors (Ekmekcioglu, 2019). Lifestyle is primarily associated with everyday physical activity, normal body mass, and a healthy diet (Szabo et al., 2018; Pignolo, 2019; Precup and Vodnar, 2019; Martău et al., 2020). To this end, there is an increasing consumer trend regarding “natural” food with added health benefits (functional food). Traditional foods’ upgrading enhances the added nutritional value of foodstuff by adding several health-related effects, like decreased risk of type 2 diabetes, obesity, hypertension, cardiovascular risk, and metabolic syndromes (Rice et al., 2019; Liu et al., 2021; Wang et al., 2021).

Cereals recently are researched concerning their ability to develop functional food because they have an increased growth (73% of the entire harvested area of the world) and provide appropriate amounts of proteins, dietary fibers, minerals, and vitamins. Wheat, part of the Poaceae family, and *Triticum* genus have a global production of approx. 765 million (M) tons (http://www.fao.org/worldfoodsituation/csdb/en/) and has the highest popularity among cereal grains. Wheat flour (WF) serves as the primary source of human food and contributes meaningfully to the human diet, containing (in 100 g) carbohydrates (71 g), proteins (13.3 g), fibers (2.3 g), minerals, and vitamins (Călinoiu and Vodnar, 2018; Charalampopoulos et al., 2002). Soybeans, part of the Fabaceae family, and the *Glycine* genus are also often used to produce functional food with an increasing world production of 352 M tons in 2017 (http://www.fao.org/faostat/en/#data). Raw soy flour (SF) is rich in nutrients, containing (in 100 g) carbohydrates (31.9 g), lipids (20.6 g), proteins (37.8 g), minerals, vitamins (https://fdc.nal.usda.gov/fdc-app.html#/food-details/174273/nutrients) and as active component isoflavones (30% daidzein and 60% genistein) (Faraj and Vasanthan, 2004; Coman et al., 2019; Hu et al., 2019). Isoflavones possess many health-promoting properties with positive effects against cancer, cardiovascular disease, hypercholesterolemia, osteoporosis, atherosclerosis, and diabetes (Faraj and Vasanthan, 2004). Cereals present a favorable substrate for lactic acid bacteria (LAB) growth. The SF and WF mixture is advantageous nutritionally, and the *β*-glucosidase enzyme from LAB can increase the aglycone content of doughs by increasing the bioactive and functional characteristics of bakery products (Thoenes, 2004; Faria et al., 2018; Zhang et al., 2018).

To naturally enhance bread quality, a convenient solution is using appropriate LAB as starter culture in single or co-cultures for bread production, with several beneficial effects (Ferraz et al., 2019; De Pasquale et al., 2020; Pontonio et al., 2020). These microorganisms can reduce breadcrumb hardness, diminish acrylamide content, increase storage time and dough elasticity (Bartkiene et al., 2017). In addition, the incorporation of LAB in dough preparation has several other important beneficial aspects, like their probiotic characteristics, which can be efficiently applied in functional food production (Min et al., 2017; Călinoiu et al., 2019a; Wang et al., 2021). The beneficial role of probiotic bacteria is primarily due to the creation of antimicrobial metabolites, elimination of enteric pathogens, the useful variation of systemic and mucosal immune behavior, and the dissolution of dietary carcinogens (Corthésy et al., 2007).

*Lactobacillus* genus taxonomically is part of the Firmicutes phylum, Bacilli class, Lactobacillales order, and Lactobacillaceae family (König et al., 2009). As energy and carbon sources, the primary substrates are carbohydrates used through the homofermentative and heterofermentative pathways. These LAB can substantially heighten different food’s antimicrobial and antioxidant effects due to biosurfactant production, which can intensify food flavors (Paucean et al., 2013; Liu et al., 2018a; Bintsis, 2018). *Lactobacillus plantarum*, an industrially relevant LAB with use in vegetable (Liu et al., 2018a; Malik et al., 2019), wine (Brizuela et al., 2019), and other food fermentations (Min et al., 2017), is a facultative heterofermentative (pentose) or homofermentative (hexose) bacterium and possesses competent adaptability to alternative microorganisms (like the yeast *Saccharomyces cerevisiae*), and environments. Fermentation of glucose with *L. plantarum* has as primary products lactic and acetic acids (Verni et al., 2019), and the fermentation of raw food, are “generally recognized as safe” (GRAS) (Brizuela et al., 2019) by the U.S. Food and Drug Administration (FDA) and has passed the Qualified Presumption of Safety (QPS) assessed by the European Food Safety Authority (EFSA) (Liu et al., 2018b). *L. casei*, an adaptive bacteria with primary usage in the dairy industry, can metabolize different carbohydrates and have heterolactic and homolactic characteristics (Calinoiu et al., 2014; Stefanovic et al., 2017). Isolated from diverse environments*, L. casei* is a rod-shaped and aciduric bacterium (Hosseini Nezhad et al., 2015). The most well-known probiotic strain in the food industry is *L. casei* ATCC 393, with many health-related positive effects, like immunity enhancement and intestinal tract regulation (Cai et al., 2019; Dimitrellou et al., 2019).

The interaction of these two LABs and their underlying mechanisms is scarce (Sieuwerts et al., 2018). Therefore, mathematical modeling is applied to anticipate the consequences of fermentation processes through the utilization of possible starter cultures, pH, oxygen content, temperature, type of substrate, accumulation of metabolites, can determine essential understanding of fermentation strategies and getting increased attention (Neysens and De Vuyst, 2005; Ricciardi et al., 2009). Nowadays, artificial neural networks (ANN) have grown in popularity when discussing the modeling process (Panerati et al., 2019). The reasoning behind the success of these models is in the properties of neural networks. Since most processes are nonlinear, the neural networks adaptability and ability to learn make them a better choice when compared to other methods. On the other hand, the main disadvantage of ANN is that, depending on the chosen training parameters, the model may have great complexity.

ANN is widely used to create mathematical models for the fermentation processes. For example, Elmeligy et al. used such tools to create a model for biobutanol production (Elmeligy et al., 2018). This study aimed to optimize the fermentation process combined with a membrane pervaporation unit and establish the optimal operating conditions. The optimization problem was solved using the dual population evolutionary algorithm. Valdez-Castro et al. used recurrent neural networks to predict the fed-batch fermentation kinetics for the bacteria *Bacillus* thuringiensis (Valdez-Castro et al., 2003). Some ANN parameter rules specified in this paper, such as the number of inputs and outputs and the number of neurons in the hidden layer, help predict and identify the appropriate fermentation process. Besides, the accuracy of the models created *via* neural networks is highlighted, with the obtained results around 2%. Liu et al. used the Levenberg-Marquardt optimization method to model ethanol production (Liu et al., 2014). They stated that this method is arguably the best method to use in modeling problems. The results obtained from their models are evaluated using the R squared value, the best value received being 0.96. Peng et al. described ANN to model a fed-batch fermentation process and a genetic algorithm (GA) to optimize the obtained model (Peng et al., 2014). The obtained mean-squared error of the model is 0.083, indicating very high accuracy. The GA was needed to find the optimal values for the inputs to maximize the production of the output. De J. C. Munanga et al. developed a global model of the lactic fermentation step of gowé by assembling blocks hosting models for bacterial growth, lactic acid production, and the drop of pH during fermentation (de J. C. Munanga et al., 2016). As concluded, the determination of kinetic parameters needs increased attention (Freire et al., 2015). The most cost- and time-saving method is by mathematical modeling (Dulf et al., 2020).

The main objective of the present work is to optimize single and co-cultures of LAB while utilizing different substrate mixtures for optimal growth and viability. The fermentation of three soy and wheat flour mixtures is studied using single cultures and co-cultures of *Lactobacillus plantarum* and *Lactobacillus casei*. It is well known, that bio-chemical processes often require a significant amount of time to obtain the optimal amount of final product. Therefore creating a mathematical model can gain important information and aids in the optimization of the process. Consequently, mathematical modeling is used to optimize the fermentation process by following these LAB’s growth kinetics and viability. Several models are created using multiple regressions and ANN with different optimization algorithms (Levenberg-Marquardt, Quasi-Newton, Scaled Conjugate Gradient, Fletcher-Powell, and Polak-Ribiere). From a modeling point of view, the considered inputs are time and wheat and soy flour concentrations, respectively. First, the model validation is done using comparisons with experimental data. Then, using the best models with the leading performance measures, optimum process values were established, like the optimal value of the final product and the optimal WF and SF concentrations.

## 2 Materials and Methods

### 2.1 Bacterial Strains and Culture Media

The present research used particular microorganisms for fermentation on different concentrations of wheat and soy-flours. The LAB used during the whole experiment were *L. plantarum* ATCC 8014 (Lp) and *L. casei* ATCC 393 (*Lc*) received from the University of Agricultural Science and Veterinary Medicine Cluj-Napoca, Romania*.* For both LAB, the used medium was MRS broth (enzyme digested casein 10 g/L, meat extract 10 g/L, yeast extract 5 g/L, glucose 20 g/L, dipotassium hydrogen phosphate 2 g/L, sodium acetate 5 g/L, di-ammonium citrate 2 g/L, magnesium sulfate 0.2 g/L, manganese sulfate 0.05 g/L, and tween 80 1.08 g/L) with a final pH of 6.4 ± 0.2 at 25°C.

#### 2.1.1 Substrate

The microbial fermentation took place on three types of wheat compositions containing 100% WF, 95% WF with 5% SF, and 90% WF with 10% SF. First, WF (type 000) was purchased from commerce and soybean (Onix variety) obtained by conventional soil tillage system with plow and 60% vegetable debris from the Agricultural Research and Development Center Turda (https://scdaturda.ro/onix/), which afterward was minced. Next, the measured flour in special 500 ml bottles was autoclaved, after which 100 ml of sterile distilled H_2_O was added. Finally, the dough was homogenized, and the appropriate amount of microorganisms was added, as previously reported (Paucean et al., 2013). Each experiment was performed in triplicate.

### 2.2 Fermentation

**FIGURE 1:**
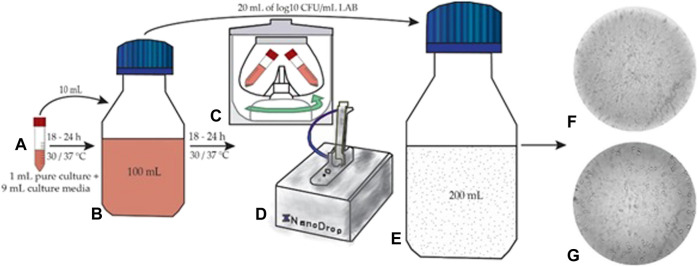
Fermentation process **(A)** microorganism activation **(B)** re-incubation **(C)** washing step **(D)** dough fermentation **(E)** viability of LAB in *Petri* plates **(F,G)** LAB under the microscope.

The first step consisted of microorganism activation on model media. The initial step was the introduction of 9 ml of MRS broth medium in special vials, after which followed the sterilization in autoclave and inoculation with 1 ml of pure *L. plantarum* or *L. casei* culture (Figure 1A). These vials with 9 ml of medium and 1 ml of pure culture were incubated at 30°C for 18–24 h. After the introduction of activated bacteria (10 ml) in 90 ml MRS broth and cell viability establishment (Figure 1B), the bottles were re-incubated for 18–24 h (Paucean et al., 2013; Teleky et al., 2020). Each step was performed under sterile conditions.

The incubated media (100 ml) with each LAB was centrifuged for 10 min at 4°C and 7,000 rpm. The supernatant was removed, and the pellet was resuspended in physiological serum (Figure 1C). This washing process was repeated twice. After this phase, the LAB concentration was measured with a UV-VIS spectrophotometer (NanoDrop 1,000; Figure 1D), and the transfer in the dough substrate was effectuated separately. The determination of bacterial concentration was performed by measuring the optical density at 600 nm (OD600) as reported in (Sieuwerts et al., 2018; Dulf et al., 2020). The dough inoculation adapted after (Gerez et al., 2012; Mitrea et al., 2019) from 100 ml media occurred with 10% (20 ml) of microorganisms at the concentration of 8 log^10^ CFU/mL (Figure 1E). The incubation time during the fermentation process was 24 h at 37°C.

### 2.3 Viability

Sample prelevation during fermentation took place at regular intervals (0, 2, 4, 6, 8, 10, and 24 h). For cell viability, the pour plate method was used for LAB and expressed using logarithmic values of the colony-forming units per mL of sample (log^10^ CFU/mL) (Călinoiu et al., 2019b). The inoculated agar plates were incubated for 48 h at 37°C, after which cell counting was performed (Figures 1F, G).

### 2.4 Modeling Methods

The first step implied using a response surface method to evaluate various operating conditions and determine the optimal parameters for the fermentation process. The multiple regression model used at the beginning considers three factors contributing to each response. Experimental data processing implies the use of polynomial equations:

$$y=a_{0}+\underset{i}{\sum }a_{i}x_{i}+\underset{i}{\sum }a_{ii}x_{i}^{2}+\underset{i,j}{\sum }a_{ij}x_{i}x_{j}+\underset{i}{\sum }a_{ii}x_{i}^{3}+\underset{i,j,k}{\sum }a_{ijk}x_{i}x_{j}x_{k}$$ (1)

where *y* is the response variable, *a*_*0*_, *a*_*i*_, *a*_*ii*_, *a*_*ij*_ are the model constant term, the coefficient of linear terms, the coefficient of quadratic and cubic terms, and the coefficient for the interaction between variables *i* and *j*, respectively *x*_*i*_ are the independent variables. The model allows the drawing of surface response curves, and through their analysis, the optimum conditions can be determined. The adequacy of the model is determined by evaluating the root mean square error, the regression coefficient (R-squared), and the F-value and *p*-value obtained from the analysis of variance (ANOVA). Matlab (R2018a) ^®^ software is used to perform the modeling, analyses, and plots.

The second research step consists in establishing a model using neural networks. The neural network model defines a family of possible equations along with a set of data and a strategy to find better rules among the possible ones based on these data. In contrast, the model form is given explicitly in multiple regression methods.

In the training stage are used 75% of experimental data. The remaining 25% of the data should be used in the model validation step. As data splitting method, the systematic stratification semideterministic method is used. The data were first ordered along the output variable dimension in increasing order. The starting point was randomly selected. Training samples were used first, followed by the testing samples.

The model is required to be as simple as possible. To achieve this, the number of layers is considered 1, and the number of neurons is maximized to 10. If the models created with these parameters have significant errors, they can be adjusted using different neural networks and optimization algorithms. As an optimization algorithm, five specific training methods are used: Levenberg-Marquardt, Quasi-Newton, Scaled Conjugate Gradient, Fletcher-Powell, and Polak-Ribiere. The obtained mathematical model has the following form:

$$y=A\cdot \left(\frac{2}{e^{B\cdot X+b_{i}}+1}-1\right)+b_{\mathrm{ii}}$$ (2)

where *y* is the response variable, *A* is a row matrix of dimension (1: number of neurons); *B* is a matrix of weights for each variable, dimension (number of variables: number of neurons); *X*: variable column matrix of dimension number of variable: 1; *b*_*i*_: column matrix of coefficients with dimension (number of neurons:1) and *b*_*ii*_ is the bias value. The adequacy of the models is determined by evaluating the root mean squared error, the regression coefficient (R-squared), and the *p*-value obtained from the analysis of variance (ANOVA). Matlab (R2018a)^®^ software is used to perform the modeling, analyses, and plots. As software is also used the tool described in (Dulf et al., 2018).

## 3 Results

### 3.1 Experimental Results

**FIGURE 2:**
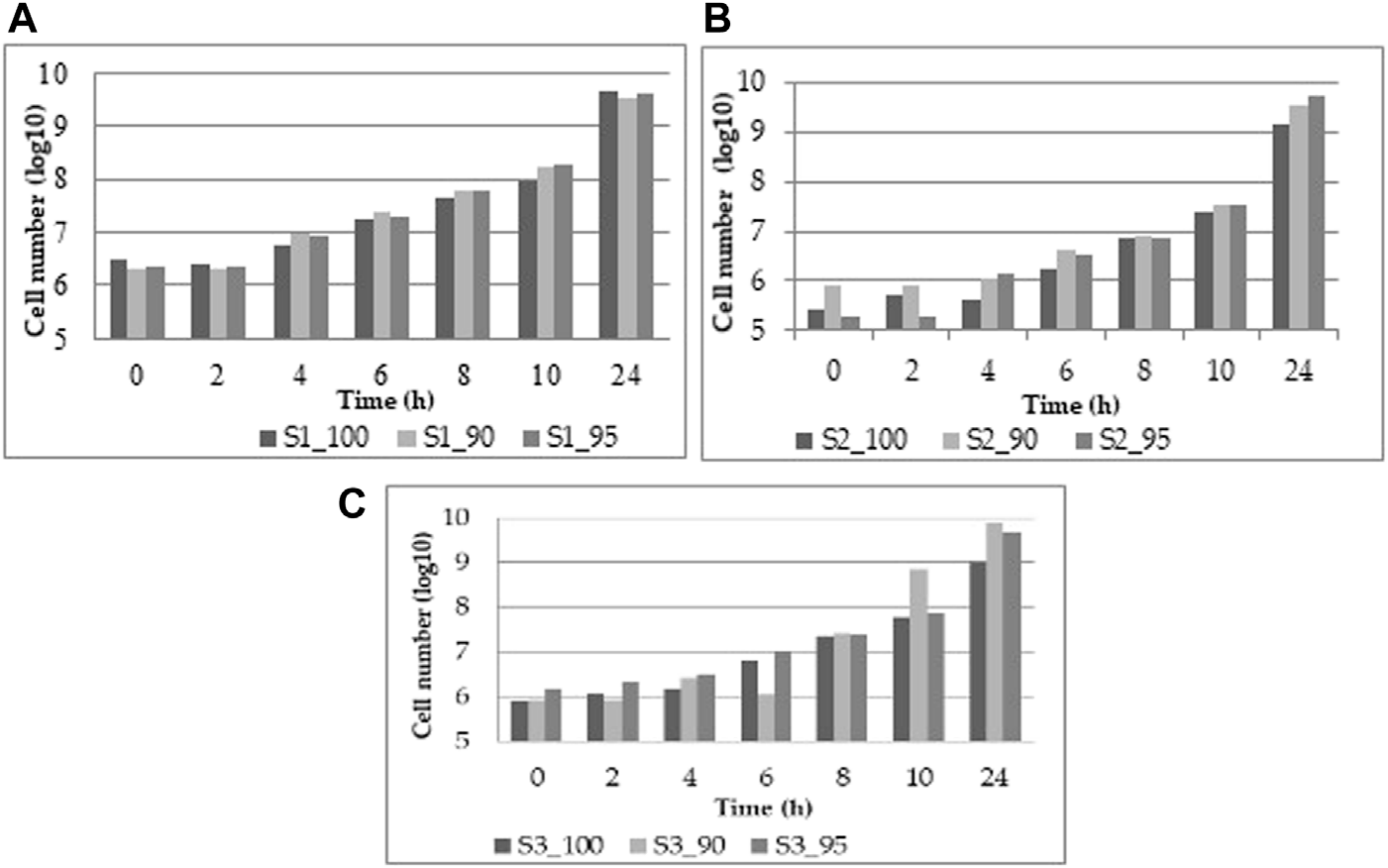
Viability results from **(A)** *L. plantarum*, **(B)** *L. casei*, **(C)** co-cultures of *L. plantarum* and *L. casei*.

The study’s purpose is to optimize fermentation by utilizing different substrate compositions of WF and SF by using LAB in single or co-cultures. In Figure 2, the viability results for the three WF and SF mixtures for the LAB *L. plantarum, L. casei,* and the co-cultures of *L. plantarum* and *L. casei* are presented*.*

The viability of the three different substrate mixtures reaches a final concentration of 9.61 ± 0.04 log CFU/mL. With L. plantarum (Figure 2A), the increase is uniform throughout the experiment. At the same time, L. casei at the beginning presents a slower growth, but the final concentration is similar to *L. plantarum* of 9.54 ± 0.11 log CFU/mL (Figure 2B). With both LAB, the viability is efficient, with the highest results of 9.67 ± 0.10 log CFU/mL (Figure 2C) The data are also available in the Appendix.

### 3.2 Multiple Regression Models

The first step is the analysis of multiple regression models derived from the three different experiments presented in the previous section. In the beginning, the used model is on *L. plantarum*, after which the *L. casei* model and the third model implied the co-cultures of *L. plantarum* and *L. casei*. The considered independent variables are x_1_ time, *x*_*2*_ concentration of wheat flour, while *x*_*3*_ represents the concentration of soybean flour.

The obtained experimental data were fitted using the four high degree polynomial models, such as the linear, interactive (2FI), quadratic, and cubic models. The test results and model summary statistics are depicted in Table 1.

**TABLE 1:** Model fitting.

Source	Sum of squares	DF	Mean square	F-Value	*p*-value
Model fitting for the *L. plantarum*					
Mean	22.823	1	22.823		
Linear	22.828	3	22.828	114.301	0.4745
2FI	22.826	5	4.5652	60.682	2.0397e-09
Quadratic	23.412	4	5.8529	172.440	6.1526e-13
Cubic	23.704	5	4.7408	283.810	2.6878e-14
Residual	1.132	19	0.060		
Total	116.720	37	10.145,025		
Model fitting for the *L. casei*					
Mean	34.525	1	34.413		
Linear	34.172	3	34.172	179.62	0.4421
2FI	34.620	5	6.924	119.25	1.5799e-11
Quadratic	34.525	4	8.631	142.97	2.644e-12
Cubic	34.962	5	6.992	198.29	3.803e-13
Residual	1.078	19	0.057		
Total	173.882	37	15.198		
Model fitting for co-cultures of *L. plantarum* and *L. casei*					
Mean	28.540	1	28.540		
Linear	28.641	3	28.641	46.728	0.2437
2FI	28.970	5	5.793	28.555	3.6923e-07
Quadratic	29.067	4	7.266	39.472	4.2459e-08
Cubic	30.350	5	6.070	54.751	4.2177e-09
Residual	3.473	19	0.183		
Total	149.041	37	12.749		

Based on the test results, the adequacy of the proposed models was estimated. To determine whether the model equations developed are significant, the analysis of variance (ANOVA) is used for the chosen models. In Table 2 the results from ANOVA for all three fermentations are presented. ANOVA also showed that the coefficient of variable *x*_*2*_ and the interaction terms *x*_*2*_ *x*_*3*_ are not significant because *x*_*2*_ and *x*_*3*_ are complementary variables. The sum of these two is 100% in each case. The resulted models are presented above.

**TABLE 2:** Regression and ANOVA results.

Regression and ANOVA Results for *L. plantarum*				
	Estimate	SE	tStat	*p*-value
x_1_	0.063390	0.039901	1.5874	0.14073
x_2_	0	0	0	0
x_3_	0	0	0	0
x_1_^2^	0.019703	0.005484	3.5924	0.00422
x_2_^2^	0.002115	0.000144	14.6900	1.4195e-08
x_3_^2^	0	0	0	0
x_1_^3^	−0.000692	0.000165	−4.1845	0.00152
x_2_^3^	−1.4844e-05	1.5918e-06	−9.3248	1.4798e-06
x_3_^3^	−4.0525e-08	1.4821e-07	−0.2734	0.78959
Root mean square error	0.129			
R-squared	0.99			
Adjusted R-squared	0.986			
F-statistic vs. constant model	284			
*p*-value	2.69e-14			
Regression and ANOVA results for *L. casei*				
	Estimate	SE	tStat	*p*-value
x_1_	0.015823	0.057974	0.2729	0.78996
x_2_	0	0	0	0
x_3_	0	0	0	0
x_1_^2^	0.026551	0.007968	3.3318	0.00669
x_2_^2^	0.002017	0.000209	9.6415	1.0635e-06
x_3_^2^	0	0	0	0
x_1_^3^	−0.000846	0.000240	−3.5201	0.00479
x_2_^3^	−1.4639e-05	2.3129e-06	−6.3292	5.6043e-05
x_3_^3^	−1.5864e-07	2.1534e-07	−0.7366	0.47674
Root mean square error	0.188			
R-squared	0.985			
Adjusted R-squared	0.98			
F-statistic vs. constant model	198			
*p*-value	3.8e-13			
Regression and ANOVA results for *L. casei*				
	Estimate	SE	tStat	*p*-value
x_1_	−0.096252	0.10279	−0.9363	0.36919
x_2_	0	0	0	0
x_3_	0	0	0	0
x_1_^2^	0.044862	0.01413	3.1751	0.00883
x_2_^2^	0.002448	0.00037	6.5992	3.8679e-05
x_3_^2^	0	0	0	0
x_1_^3^	−0.001450	0.00042	−3.4014	0.00591
x_2_^3^	−1.8861e-05	4.1009e-06	−4.5993	0.00076
x_3_^3^	2.614e-07	3.8182e-07	0.68462	0.50775
Root mean square error	0.333			
R-squared	0.948			
Adjusted R-squared	0.931			
F-statistic vs. constant model	54.8			
*p*-value	4.22e-09

Case 1
$$y=0.00069266x_{1}^{3}-1.4844e-05x_{2}^{3}-4.0525e-08x_{3}^{3}+0.019703x_{1}^{2}+0.0021154x_{2}^{2}+0.06339x_{1}$$ (3)

Case 2
$$y=-0.00084662x_{1}^{3}-1.4639\text{e}-05x_{2}^{3}-4.0525\text{e}-08-1.5864\text{e}-07x_{3}^{3}+0.026551x_{1}^{2}+0.0020173x_{2}^{2}+0.015823x_{1}$$ (4)

Case 3
$$y=-0.0014505x_{1}^{3}-1.8861\text{e}-05x_{2}^{3}+2.614\text{e}-07x_{3}^{3}+0.044862x_{1}^{2}+0.0024482x_{2}^{2}-0.096252x_{1}$$ (5)

**FIGURE 3:**
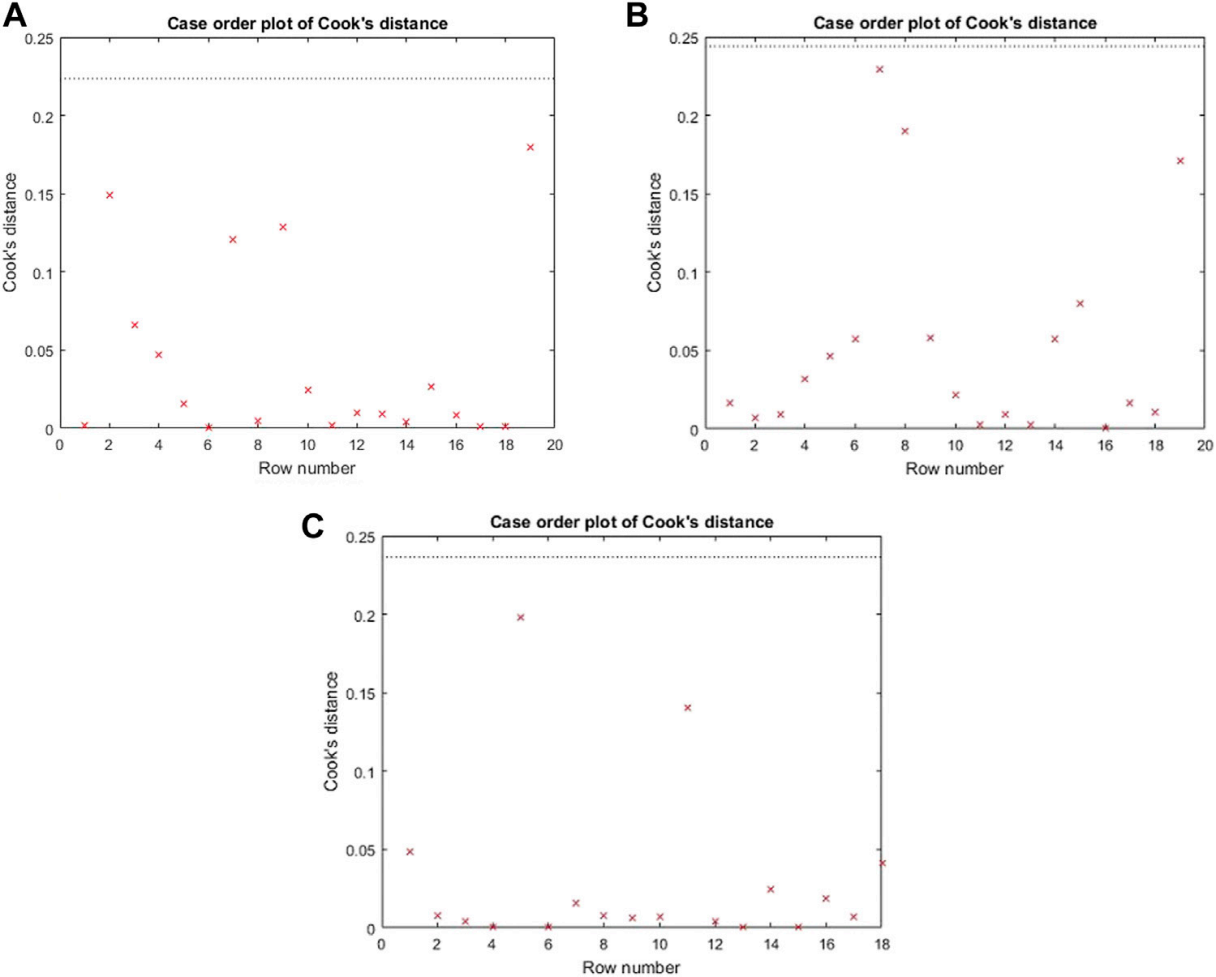
Cook’s distance for **(A)** *L. plantarum*; **(B)** *L. casei*; **(C)** co-cultures of *L. plantarum* and *L. casei*.

**FIGURE 4:**
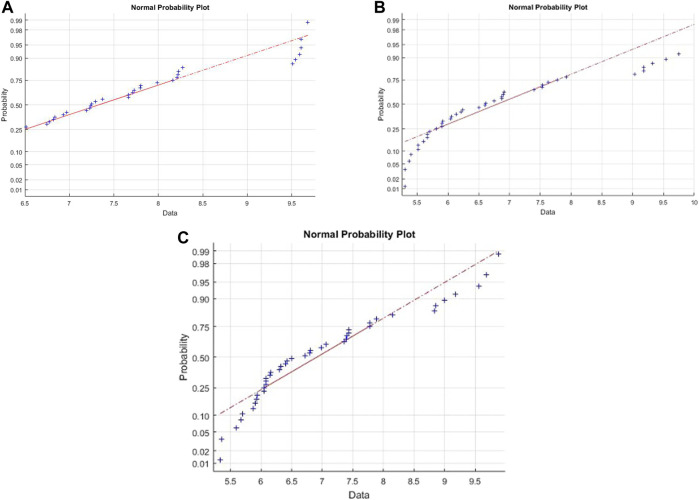
Normal probability for **(A)** *L. plantarum*; **(B)** *L. casei*; **(C)** co-cultures of *L. plantarum* and *L. casei*.

The Cook’s distance and the normal probability plot for the obtained models are presented in Figures 3, 4, where subfigures 1) represent the results for *L. plantarum*, 2) are for *L. casei*, while 3) is for the co-culture of *L. plantarum* and *L. casei*. Since there were no significant deviations from the straight line in the normal probability plot, it can be concluded that the standardized residuals of the model have a normal distribution. The Cook’s distance implied that the outliers were not present among the data fitted by the regression model.

**FIGURE 5:**
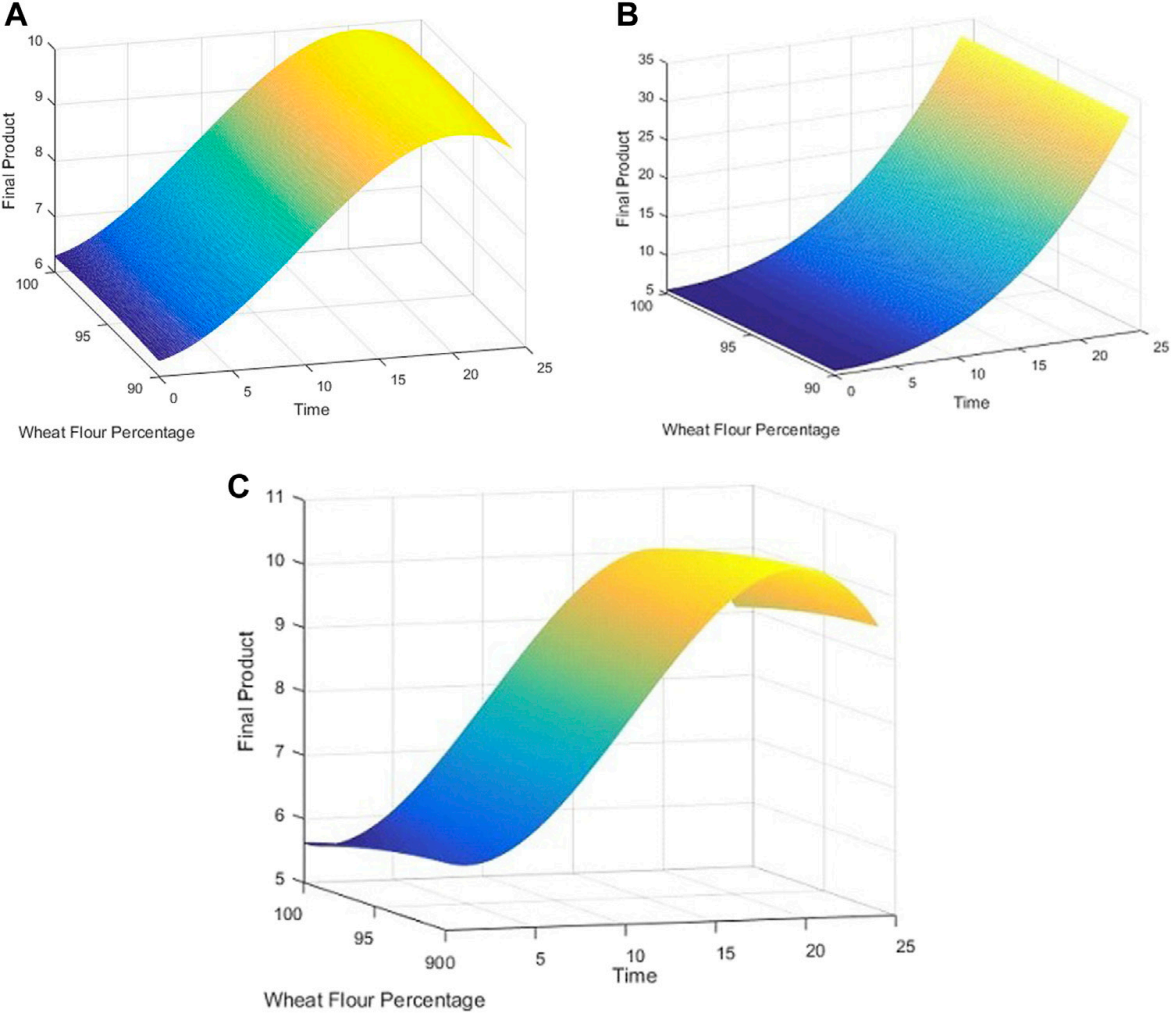
3D plots for **(A)** *L. plantarum*; **(B)** *L. casei*; **(C)** co-cultures of *L. plantarum* and *L. casei*.

The three-dimensional plots are used to present the impact of analyzed factors on the fermentation by utilizing different substrate compositions of WF and SF by using LAB in single or co-cultures. The effects of fermentation time, the concentration of wheat flour, and the concentration of soybean flour are depicted in Figure 5, using the same labeling as in previous figures: 1) for the results for *L. plantarum*, 2) for *L. casei* and 3) for the co-culture of *L. plantarum* and *L. casei*. Based on the presented plots, the relationship between the response and factors can be considered. The extraction time exhibited a linear, quadratic (in the second case) and a cubic effect, reaching a maximum value and later decreasing in value. The concentration of wheat flour effect presents a slight linear increase. The concentration of soybean flour effect presents a slight linear decrease.

### 3.3 ANN Models

Although the regression model exhibits good results, being a more advanced modeling tool, ANN models are also developed. The best optimization algorithm and the optimum number of neurons for the neural network are obtained using experimental data. Analyzing the mean squared error in each case are obtained as competitive the models developed with the Levenberg-Marquardt, Quasi-Newton and Fletcher-Powell algorithm with 6, 8 and 10 neurons. The obtained mean squared errors are: 0.0265 for Levenberg-Marquardt with 6 neurons, 0.0306 for Levenberg-Marquardt with 8 neurons, 0.0377 for Quasi-Newton with 8 neurons, 0.0439 for Quasi-Newton with 10 neurons and 0.0364 for Fletcher-Powell with 10 neurons.

**FIGURE 6:**
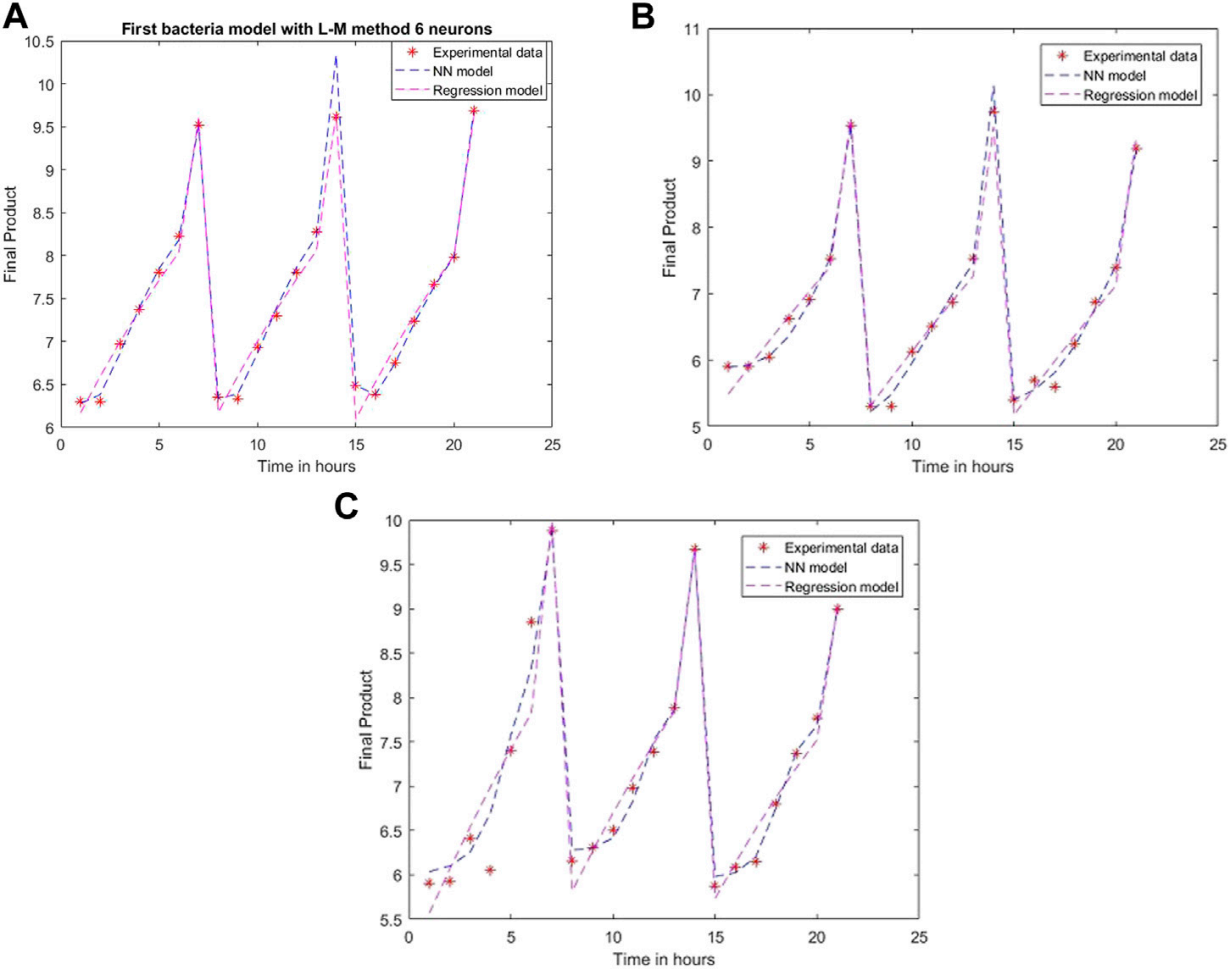
Model results with the Levenberg-Marquardt method and six neurons for **(A)** *L. plantarum*; **(B)** *L. casei*; **(C)** co-cultures of *L. plantarum* and *L. casei*.

**FIGURE 7:**
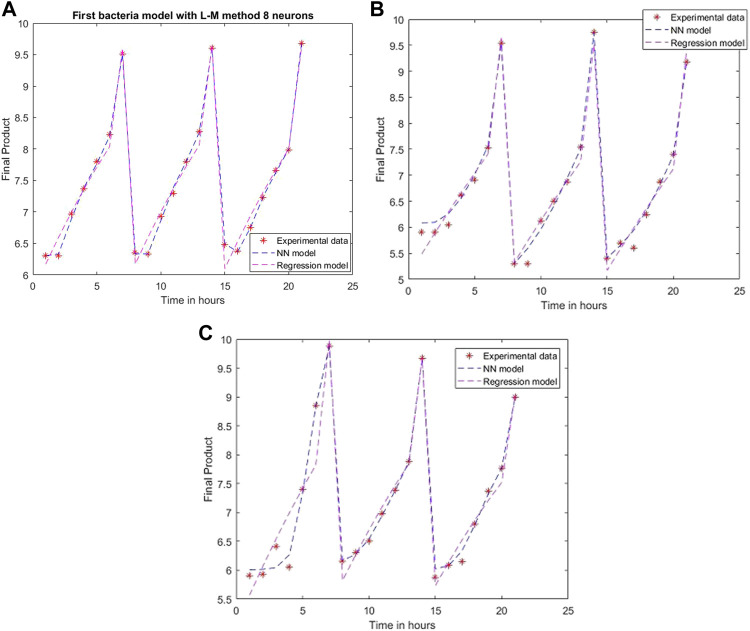
Model results with Levenberg-Marquardt method and eight neurons for **(A)** *L. plantarum*; **(B)** *L. casei*; **(C)** co-cultures of *L. plantarum* and *L. casei*.

**FIGURE 8:**
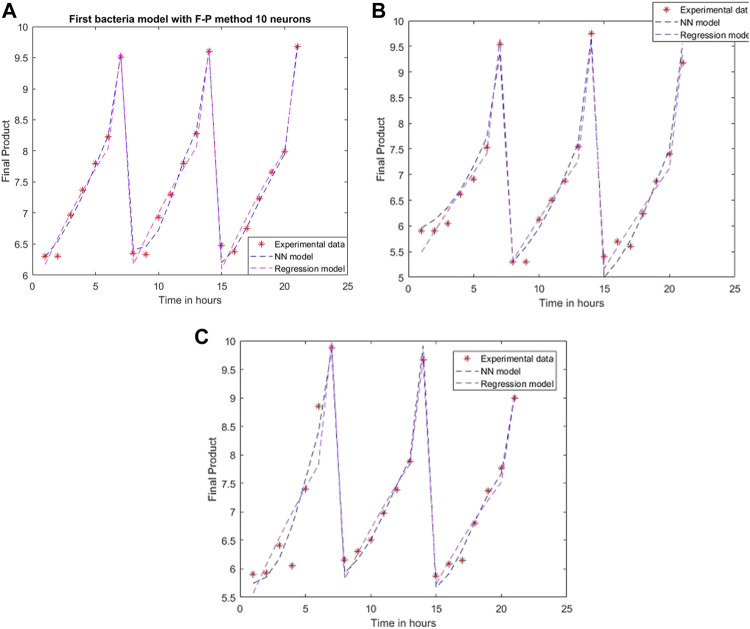
Model results with Fletcher-Powell method and 10 neurons for **(A)** *L. plantarum*; **(B)** *L. casei*; **(C)** co-cultures of *L. plantarum* and *L. casei*.

An ANN model is established for each experimental setting case with different training methods and several neurons. The comparison between the 2D simulation results of ANN models with Levenberg-Marquardt training methods with different neuron numbers and the multiple linear regression from the models mentioned above are presented in Figures 6–8, with the labeling of subplots as follows: 1) for the results for *L. plantarum*, 2) for *L. casei* and 3) for the co-culture of *L. plantarum* and *L. casei*. The experimental data of cell viability for LAB are presented with stars and are expressed using logarithmic values of the colony-forming units per mL of sample (log10 CFU/mL). For a more accurate model validation, one more dataset was realized, with a smaller sampling period. Using more neurons improves the model’s performance, as presented in Figure 7. The disadvantage is the model complexity. Some of the matrices will grow in size from 6 to 8.

Using the Fletcher-Powel method, the obtained models are more complex, having matrices with 10 rows and 10 columns. The performance of the models is worse than the achievements of the models created via Levenberg-Marquardt, being 0.0113, 0.0441, and 0.0513 in the considered cases. The results are plotted in Figure 8.

**FIGURE 9:**
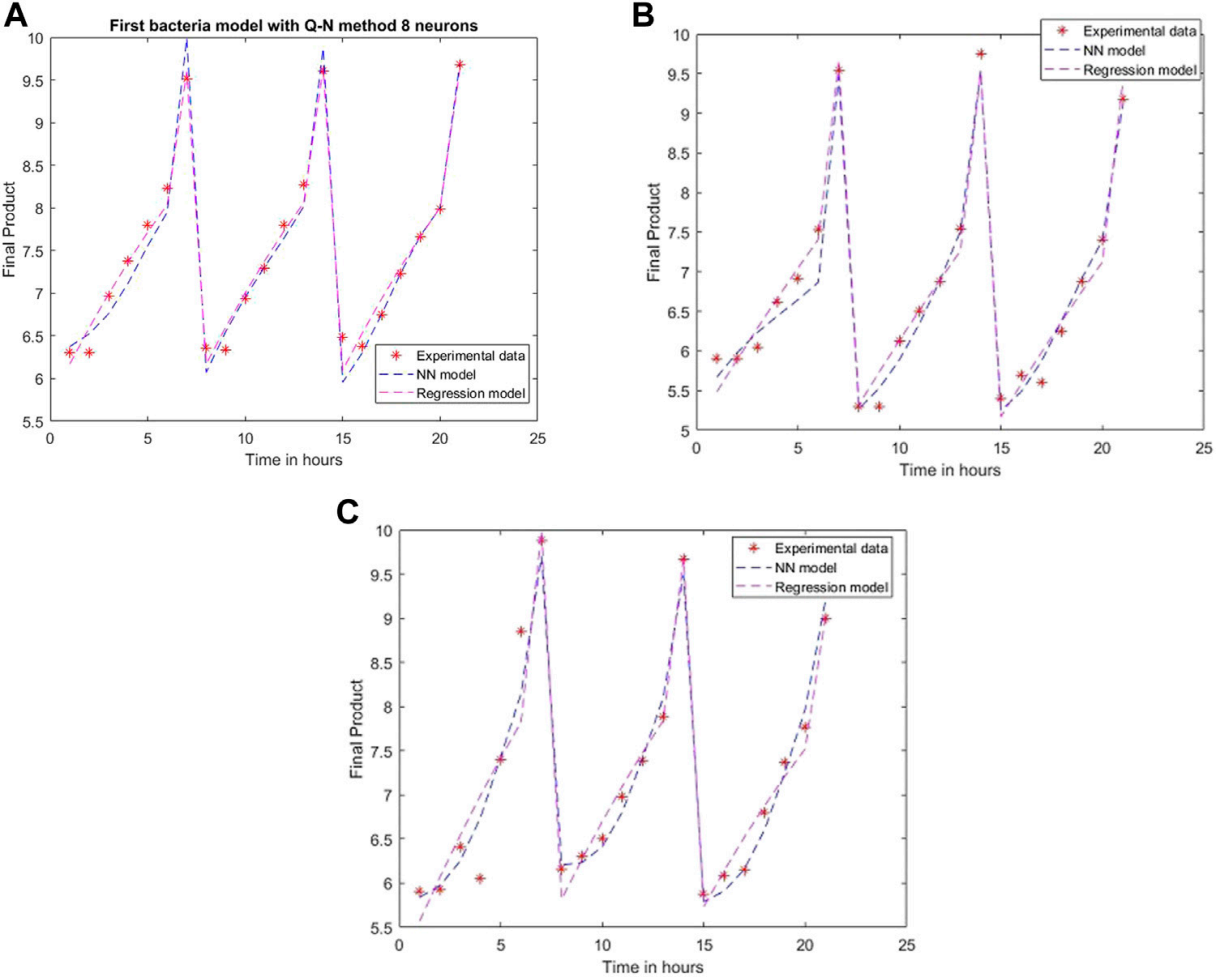
Model results with the Quasi-Newton method and eight neurons for **(A)** *L. plantarum*; **(B)** *L. casei*; **(C)** co-cultures of *L. plantarum* and *L. casei*.

Using the Quasi-Newton method as a training network, the created models lead to the errors: 0.0523, 0.0467, and 0.0627, with the simulation results presented in Figure 9. Since it has the same number of neurons as the Levenberg-Marquardt method, it is just as complex, but the performances are more accurate for the model created with Levenberg-Marquardt.

The mathematical models obtained in these three case studies have the form:

$$y=A\cdot \left(\frac{2}{e^{B\cdot X+b_{i}}+1}-1\right)+b_{ii}$$ (6)

The *L. plantarum* case model:

$$b_{i}=\left(\begin{array}{c} 2.97\\ 1.8\\ 1.58\\ -0.03157\\ -0.187\\ 2.53\\ 2.3\\ 3.73 \end{array}\right);B=\left(\begin{array}{ccc} -1.49 & -1.97 & -0.73\\ -1.78 & -0.88 & -1.84\\ -1.78 & -1.79 & -0.36\\ -1.61 & -2.02 & 1.01\\ 3.4 & 0.16 & 0.23\\ 2.62 & 0.91 & 0.68\\ 3.1 & 1.08 & 1.39\\ 3.21 & 0.99 & 0.94 \end{array}\right);$$ (7)

$$A=\left(\begin{array}{cccccccc} 0.08 & 0.39 & 0.27 & -0.025 & 0.86 & 0.9 & 0.56 & -1.98 \end{array}\right);b_{\mathrm{ii}}=0.563.$$ (8)

The *L. casei* case model:

$$b_{i}=\left(\begin{array}{c} 2.81\\ -1.88\\ 1.044\\ 0.22\\ 0.47\\ -1.86\\ -2.64\\ -2.81 \end{array}\right);B=\left(\begin{array}{ccc} -2.49 & 1.21 & -1.12\\ 2.7 & -0.82 & 0.85\\ -0.78 & -0.84 & -2.61\\ 0.16 & -2.58 & 0.49\\ 1.91 & 0.44 & 0.51\\ -1.77 & -1.63 & -0.911\\ -0.338 & -2.15 & -0.3\\ -2.021 & -1.119 & 1.18 \end{array}\right)$$ (9)

$$A=\left(\begin{array}{cccccccc} -0.17 & 0.33 & 0.62 & 0.08 & 0.79 & -0.36 & 1.36 & 0.17 \end{array}\right);b_{\mathrm{ii}}=1.0356.$$ (10)

The co-cultures of *L. plantarum* and *L. casei* case model:

$$b_{i}=\left(\begin{array}{c} 2.82\\ -1.99\\ -0.49\\ 0.77\\ 1.1\\ -0.27\\ -2.26\\ -1.71 \end{array}\right);B=\left(\begin{array}{ccc} -2.88 & 1.02 & 0.9\\ 0.2 & 1.68 & -2.24\\ 2.24 & 1.69 & -0.8\\ -6.3 & 3.09 & 0.43\\ -0.66 & -0.27 & 2.69\\ 2.12 & 1.12 & -0.2\\ -1.12 & 1.51 & -1.15\\ -3.07 & -2.31 & 1.44 \end{array}\right);$$ (11)

$$A=\left(\begin{array}{cccccccc} 1.38 & 0.17 & -0.37 & -0.82 & 0.39 & 0.98 & 0.05 & -0.35 \end{array}\right);b_{\mathrm{ii}}=-0.72.$$ (12)

**FIGURE 10:**
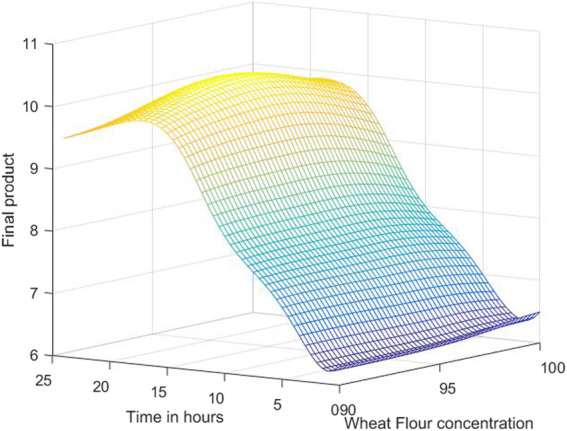
3D model simulation results with the Levenberg-Marquardt method and eight neurons for *L. plantarum*.

**FIGURE 11:**
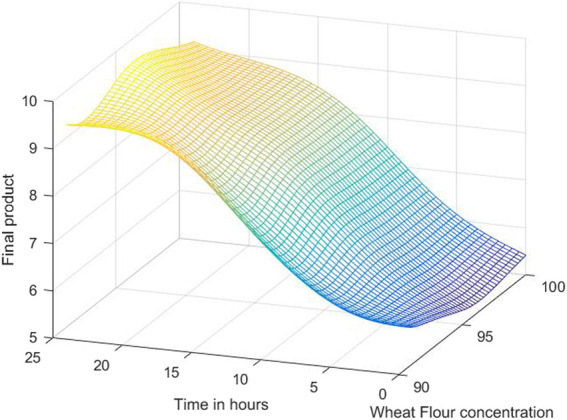
3D model simulation results with the Levenberg-Marquardt method and eight neurons for *L. casei*.

**FIGURE 12:**
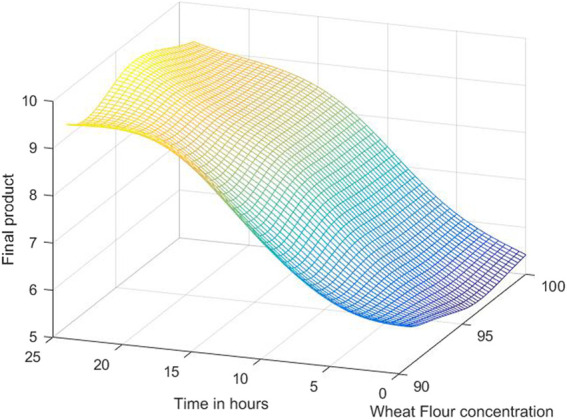
3D model simulation results with the Levenberg-Marquardt method and eight neurons for co-cultures of *L. plantarum* and *L. casei*.

The 3D simulation results of these models are presented in Figures 10, 11, 12. Figure 10 presents the 3D model simulation results with the Levenberg-Marquardt method with eight neurons for *L. plantarum;* Figure 11 is for results with the Levenberg-Marquardt method with eight neurons for *L. casei*, while Figure 12 show the results with the Levenberg-Marquardt method with eight neurons for co-cultures of *L. plantarum* and *L. casei*.

With the use of the 3D plots and the use of the models, an approximation of the optimal concentration is found by searching the highest point. For the first process, the optimum value of the final product is 10.3076 obtained for 97.7083% concentration of WF and 19 h of fermentation.

In the case of the second process, the optimum value is 9.7571, with a concentration of 95.625% WF in 21 h.

The third process has a maximum amount of 12.2755, with 97.7083% WF in 19 h. Under these optimal conditions, repeated validation experiments are performed, obtaining close values to the predicted ones.

## 4 Discussions

### 4.1 Regression Models Analysis

From the ANOVA test of the regression model for *L. plantarum,* the *p*-value is 2.69·10^−14^, which means that the model is significant. R squared has the value of 0.99, showing that the regression model fits well for the first bacterium. The root-mean-squared error has the value of 0.129, proving that the quadratic response for this case fits the results obtained in the experiment.

In the case of the second bacterium, *L. casei*, the *p*-value is 3.8·10^−13^ with the R squared value 0.985. The root mean squared error is 0.188. The values are not so different from the previous results giving almost as good performance as in the first case.

For the final case, with the co-cultures of *L. plantarum* and *L. casei*, the *p*-value is 4.22·10^−9^ with R squared value of 0.948. The root means squared error is 0.333. Out of the three cases, this one presents the less agreeable performances, but still a good result. The results obtained with regression are good for all three cases, with mean squared error under the accepted values.

### 4.2 ANN Models Analysis

The performance of the developed ANN models is evaluated by mean squared error. The first developed ANN models were with the Levenberg-Marquardt method with six neurons on each layer. The obtained mean squared error for the *L. plantarum* is 0.028, for *L. casei* 0.0176, and the mixed culture of this two is 0.0418. Increasing the number of neurons on each layer can lead to better performances. That was the reason for developing models with eight neurons. The obtained mean squared errors are for the first case 0.0014, for the second case 0.0185, and 0.0124 for the third case. However, a great influence on the results has the used optimization algorithm. For example, the performance of the models with 10 neurons with the Fletcher-Powel method is worse than the achievements of the models created *via* Levenberg-Marquardt with 8 neurons, being 0.0113, 0.0441, and 0.0513 in the considered cases instead of 0.0014, 0.0185, and 0.0124. Using the Quasi-Newton method with 8 neurons as a training network, the created models lead to the errors: 0.0523, 0.0467, and 0.0627.

### 4.3 Comparison of Regression and Different ANN Models

The synthesis of the considered ANN models performance measures (Table 3), compared with the results obtained with the regression method, highlights the superiority of the ANN models. The best-considered model for this process (with all three used bacterium) is obtained with the Levenberg-Marquardt method using eight neurons.

**TABLE 3:** Performance measures for the models created.

Performance Measures for the Models Created for *L. plantarum*			
Method/Number of Neurons	Mean Squared Error	R-value	*p*-Value
Regression	0.1900	0.97700	8.45e-12
Levenberg-Marquardt/6	0.0280	0.99150	2.82e-18
Levenberg-Marquardt/8	0.0014	0.99940	2.60e-29
Quasi-Newton/8	0.0523	0.98250	2.44e-15
Fletcher-Powell/10	0.0113	0.99552	1.30e-20
Performance measures for the models created for *L. casei*			
Method/Number of neurons	Mean squared error	R-value	*p*-value
Regression	0.2540	0.9730	3.38e-11
Levenberg-Marquardt/6	0.0176	0.9955	6.59e-21
Levenberg-Marquardt/8	0.0185	0.9957	4.26e-21
Quasi-Newton/8	0.0467	0.9878	8.21e-17
Fletcher-Powell/10	0.0441	0.9883	5.66e-17
Performance measures for the models created for co-cultures of *L. plantarum* and *L. casei*			
Method/Number of neurons	Mean squared error	R-value	*p*-value
Regression	0.4110	0.9210	9.42e-8
Levenberg-Marquardt/6	0.0418	0.9871	1.38e-16
Levenberg-Marquardt/8	0.0124	0.9961	1.79e-21
Quasi-Newton/8	0.0627	0.9796	1.06e-14
Fletcher-Powell/10	0.0513	0.9838	1.21e-15

For the first case, with *L. plantarum,* the best results are obtained with the Levenberg-Marquardt method using 8 neurons. In the case of the *L. casei*, the model with Levenberg-Marquardt with 6 and 8 neurons presents similar performances, but because the best model in most cases is Levenberg-Marquardt with 8 neurons, the decision is to use the same parameters in all cases. As a result, the performance for the two methods differs by an insignificant amount. The co-culture of *L. plantarum* and *L. casei* leads to the same conclusion: the best model is obtained with Levenberg-Marquardt with 8 neurons. The superiority of the ANN models is proved by the 100 times smaller mean squared error in almost every case.

### 4.4 Optimization Results

The optimum process conditions can be found using a standard optimization algorithm for each developed model. For the fermentation process with *L. plantarum*, the optimum value of the final product concentration could be 10.3076 log CFU/mL, obtained for 97.7083% concentration of WF and 19 h of fermentation. In the case of *L. casei* bacteria, the optimum value is 9.7571 log CFU/mL, with a concentration of 95.625% WF in 21 h. The case of co-culture of these two bacteria leads to the best results. The optimum value is 12.2755 log CFU/mL, with 97.7083% WF in 19 h. These results are of great interest for such fermentation processes. Using co-cultures of different bacteria can improve the process performances, as proved in this research.

## 5 Conclusion

The tests with three types of substrate compositions evaluated the efficiency and ability of two LAB, *L. plantarum* ATCC 8014 and *L. casei* ATCC 393, to metabolize various carbohydrates and analyze their effectiveness in single and co-cultures for dough fermentation. *L. plantarum* presented the most efficient growth dynamics and viability, reaching a concentration above 9.61 log^10^ CFU/mL. Although *L. casei* had a prolonged growth dynamic in the first 10 h, at 24 h, the cell concentration was around 9.54 ± 0.11 log CFU/mL. Together, the two LAB grew in harmony, good cell viability, and efficient growth dynamics.

Process optimization was performed by mathematical modeling. The presented results prove the superiority of the models created with neural networks compared to classical multiple regression analysis. Comparing all the different training methods, the Levenberg-Marquardt process was found to be the most dominant and had the best performances while having a relatively small number of neurons used to create the model. The performance measures obtained with eight neurons for the case of *L. plantarum* are: Mean squared error 0.0014, R-value 0.9994, *p*-value 2.6e-21. For the second case study, *L. casei*, the results are: Mean squared error 0.0185, R-value 0.9957, *p*-value 4.26e-21. The third case, co-cultures of *L. plantarum* and *L. casei,* leads to a mean squared error of 0.0124, R-value 0.9961, *p*-value 1.79e-21. These results far outweigh the results obtained by the regression method with a mean squared error of 0.129 for the first case, 0.188 for the second case, and 0.333. The obtained models can be used safely to predict or to optimize the process. The process optimization concluded that the best results could be obtained for the co-culture of *L. plantarum* and *L. casei*. The optimum value, in this case, is 12.2755 log CFU/mL, with 97.7083% WF in 19 h.

## Data Availability

The raw data supporting the conclusion of this article will be made available by the authors, without undue reservation.

## Appendix

**Table:**

***L. plantarum***	**Time (h)**	**Cell number (log**_**10**_ **)**								
		**Wheat flour 100%**			**Wheat flour 90%, Soybean flour 10%**			**Wheat flour 95%, Soybean flour 5%**		
		Exp1	Exp2	Exp3	Exp1	Exp2	Exp3	Exp1	Exp2	Exp3
	0	6.447	6.519	6.462	6.255	6.342	6.322	6.342	6.362	6.322
	2	6.362	6.380	6.398	6.301	6.301	6.279	6.322	6.342	6.301
	4	6.672	6.748	6.813	7.004	6.898	6.954	6.954	6.934	6.914
	6	7.230	7.241	7.220	7.371	7.373	7.375	7.294	7.281	7.307
	8	7.633	7.667	7.698	7.786	7.810	7.799	7.773	7.826	7.800
	10	7.986	7.969	8.003	8.256	8.227	8.195	8.284	8.275	8.266
	24	9.792	9.519	9.681	9.362	9.623	9.519	9.230	9.806	9.602
***L.casel***	**Time (h)**	**Cell number (log**_**10**_ **)**								
		**Wheat flour 100%**			**Wheat flour 90%, Soybean flour 10%**			**Wheat flour 95%, Soybean flour 5%**		
		Exp1	Exp2	Exp3	Exp1	Exp2	Exp3	Exp1	Exp2	Exp3
	0	5.699	5.477	5.000	5.845	5.903	5.954	5.301	5.301	5.301
	2	5.477	5.699	5.845	5.845	5.954	5.903	5.477	5.000	5.301
	4	5.477	5.602	5.699	6.000	6.041	6.079	6.146	6.146	6.114
	6	6.204	6.255	6.279	6.556	6.613	6.663	6.519	6.505	6.491
	8	6.875	6.869	6.875	6.968	6.914	6.845	6.881	6.875	6.863
	10	7.386	7.396	7.407	7.566	7.525	7.479	7.554	7.517	7.537
	24	7.161	7.179	7.196	7.533	7.550	7.542	7.752	7.772	7.731
***L. plantarum+ L. casei***	**Time (h)**	**Cell number (log**_**10**_ **)**								
		**Wheat flour 100%**			**Wheat flour 90%, Soybean flour 10%**			**Wheat flour 95%, Soybean flour 5%**		
		Exp1	Exp2	Exp3	Exp1	Exp2	Exp3	Exp1	Exp2	Exp3
	0	5.845	5.845	5.903	5.954	5.903	5.845	6.255	6.176	6.041
	2	6.114	6.079	6.000	6.000	5.954	5.845	6.380	6.204	6.301
	4	6.204	6.146	6.041	6.505	6.279	6.415	6.505	6.447	6.556
	6	6.820	6.799	6.771	7.041	7.049	7.064	6.968	6.982	6.996
	8	7.384	7.352	7.318	7.352	7.401	7.446	7.378	7.387	7.393
	10	7.751	7.793	7.773	7.838	7.857	7.848	7.881	7.906	7.854
	24	7.013	6.996	6.973	7.877	7.888	7.866	7.667	7.678	7.672
